# Health-Related Quality of Life of Lebanese Women With Breast Cancer: Protocol for a Prospective Cohort Study

**DOI:** 10.2196/27893

**Published:** 2021-11-23

**Authors:** Rana El Haidari, Amelie Anota, Linda Abou-Abbas, Virginie Nerich

**Affiliations:** 1 INSERM (French Institut of Health and Medical Research), EFS BFC (Etablissement français du sang Bourgogne Franche-Comté), UMR1098 (Interactions Greffon-Hôte-Tumeur/Ingénierie Cellulaire et Génique), University of Bourgogne Franche-Comté, RIGHT Interactio Besancon France; 2 French National Platform Quality of Life and Cancer Besançon France; 3 Neuroscience Research Center Faculty of Medical Sciences Lebanese University Hadath Lebanon; 4 Department of Pharmacy, University Hospital of Besançon Besançon France

**Keywords:** breast cancer, cohort, health-related quality of life, Lebanese women, prospective

## Abstract

**Background:**

In the past few decades, Lebanon has witnessed a significant increase in the incidence rates of women diagnosed with breast cancer. This increase, which is associated with the advancements in treatment modalities, emphasizes the need to evaluate the health-related quality of life (HRQoL) of women with breast cancer and to compare its patterns before and after breast-conserving surgery (BCS).

**Objective:**

This study aims to describe changes in HRQoL according to body image pre- and post-BCS and just before initiation of adjuvant therapy in newly diagnosed patients with breast cancer in Lebanon.

**Methods:**

A prospective cohort study targeting Lebanese women newly diagnosed with breast cancer and who have an indication for BCS will be conducted in 2 health care facilities. Baseline characteristics and clinical data will be collected. The European Organization for Research and Treatment of Cancer Quality-of-Life cancer-specific and breast cancer–specific questionnaires will be used to assess HRQoL. The outcomes will be measured at baseline and 1 day after breast surgery. The primary outcome will be the body image dimensions of the Quality-of-Life breast cancer–specific questionnaire. Statistical analyses will include descriptive statistics, paired 2-tailed *t* test, and stepwise multiple regression. A total of 120 patients will be required.

**Results:**

A total of 120 patients were enrolled in the study. Future outcomes will be published in professional peer-reviewed health-related research journals.

**Conclusions:**

This study is strengthened by its follow-up nature, allowing us to draw conclusions about causality. The results of this study will identify the most affected components of HRQoL, as well as the factors that could play a role in improving HRQoL among women undergoing BCS. The findings of this study will help decision makers, physicians, and social workers to design a comprehensive program with multidisciplinary components for the management and care of patients with breast cancer in Lebanon.

**International Registered Report Identifier (IRRID):**

DERR1-10.2196/27893

## Introduction

### Background

Approximately 2.1 million women were newly diagnosed with breast cancer in 2018 worldwide [1]. According to Global Cancer Observatory estimates, 684,996 breast cancer–related deaths occurred in 2020 [2]. In the Arab World, the incidence of breast cancer represents 14%-42% of all cancers in women [3]. This wide range is attributed to the capability of each country to detect, monitor, and treat patients with breast cancer [4]. For example, Kuwait, Bahrain, and Qatar can be classified as high-incidence areas with >40% of all female cancers [5]. Meanwhile, the United Arab Emirates, Saudi Arabia, and Oman are classified as low-incidence areas [6]. Lebanon, in particular, is a small Arabic middle-income country with a prominent breast cancer incidence [7]. A significant number of Lebanese women are diagnosed with an incidence rate that has been on the rise over the years, going from a reported 1451 cases in 2005 to 3219 cases in 2018 [8-10]. Approximately 920 (10%) breast cancer–related deaths occurred in 2018 [1].

Breast-conserving surgery (BCS) is one of the most recommended treatments for early-stage breast cancer [11,12]. According to El Saghir et al [4], BCS in Lebanon increased from 48% between 1997 and 2002 to 64% between 2002 and 2010, with a corresponding decrease in the total mastectomy rate from 51% to 36%.

Despite the increase in BCS, this treatment carries an array of side effects and uncomfortable physical symptoms as well as psychological disturbances, such as fear of recurrence, feelings of decreased femininity and attractiveness, and depression. The experience of breast cancer has a prevailing effect on female body image, which varies according to the clinical features and phases of the disease [13]. Unfortunate body image perceptions have the potential to negatively impact the physical and psychological functioning of patients with breast cancer and subsequently their health-related quality of life (HRQoL) [14].

Body image is defined as women’s perception and feelings about their body and their self-observation, self-esteem, social interaction, and belief [15]. Han et al [16] found that patients with breast cancer who had better conceptualization of their body image better managed with cancer. Furthermore, fatigue is one of the most common and disabling HRQoL symptoms of cancer among women successfully treated for breast cancer [17]. Fatigue is defined as a general feeling of debilitating tiredness or loss of energy. Fatigue can be associated with several symptoms, such as pain, sleep disturbance, and depression [18,19]. Thus, the impact of body image and fatigue on HRQoL can be extensive, reducing the patient’s engagement in work and personal and social activities [20]. With the intention of supporting patients who adapt to their illness and report positive mental health states, several studies have been performed to identify psychological resources that predict better outcomes. One of these resources is habitual or dispositional optimism, a personality trait that describes the degree to which a person generally expects positive outcomes [21,22]. Dispositional optimism is associated with HRQoL [23,24].

Robust scientific data on the HRQoL of patients with breast cancer postactive treatment in Lebanon, especially those who experienced BCS, are still scarce. Only 2 single-center studies with cross-sectional designs were conducted in Lebanon [25,26]. Data of the first study collected from 89 participants between 2009 and 2010 showed that younger, single, and better-educated participants who were diagnosed for <30 months, who had no metastasis, and who paid <US $450 per month on medical expenses had better HRQoL. The second study conducted among 150 female patients with breast cancer diagnosed between January 2009 and March 2014 showed that participants who were Iraqi, had stage 4 disease, had a monthly household income <US $1000, or had received chemotherapy exhibited significantly lower HRQoL. Impaired quality of life (QoL) was also reported in patients with worse psychological well-being [26]. The results of this study cannot be generalized because of the lack of a well-representative sample and the small sample size of patients [27-30]. In addition, this study did not compare the QoL of the same individuals at several time points but rather compared different participants with various times elapsed since diagnosis.

### Objectives

This study aims to describe changes in the HRQoL according to body image pre- and post-BCS and just before initiation of adjuvant therapy in newly diagnosed patients with breast cancer in Lebanon. The secondary objectives are (1) to assess changes in the HRQoL according to fatigue and optimism and pessimism between pre- and post-BCS and (2) to identify sociodemographic and clinical factors associated with changes in the HRQoL.

## Methods

### Study Design

To meet the objectives of the study, a prospective study must be conducted.

### Characteristics of Participants

#### Inclusion Criteria

Only the patients who met the inclusion criteria were invited to participate in this study.

Patient inclusion criteria are women aged ≥18 years; recently diagnosed with invasive early breast cancer of stages 1, 2a, and 2b who are scheduled to undergo breast surgery as primary treatment; without a history of another type of cancer or metastasis; with an absence of other medical or psychiatric conditions; with no previous chemotherapy or radiotherapy; able to read and write the Arabic language; and able to sign informed consent.

#### Exclusion Criteria

Patients were excluded if they met any of the following exclusion criteria: women who are pregnant or breastfeeding, women who were treated with neoadjuvant chemotherapy, women who had bilateral breast cancer, and women with an active infection or other underlying serious conditions that may prevent the patient from receiving surgery.

### Proceedings

**Figure 1:**
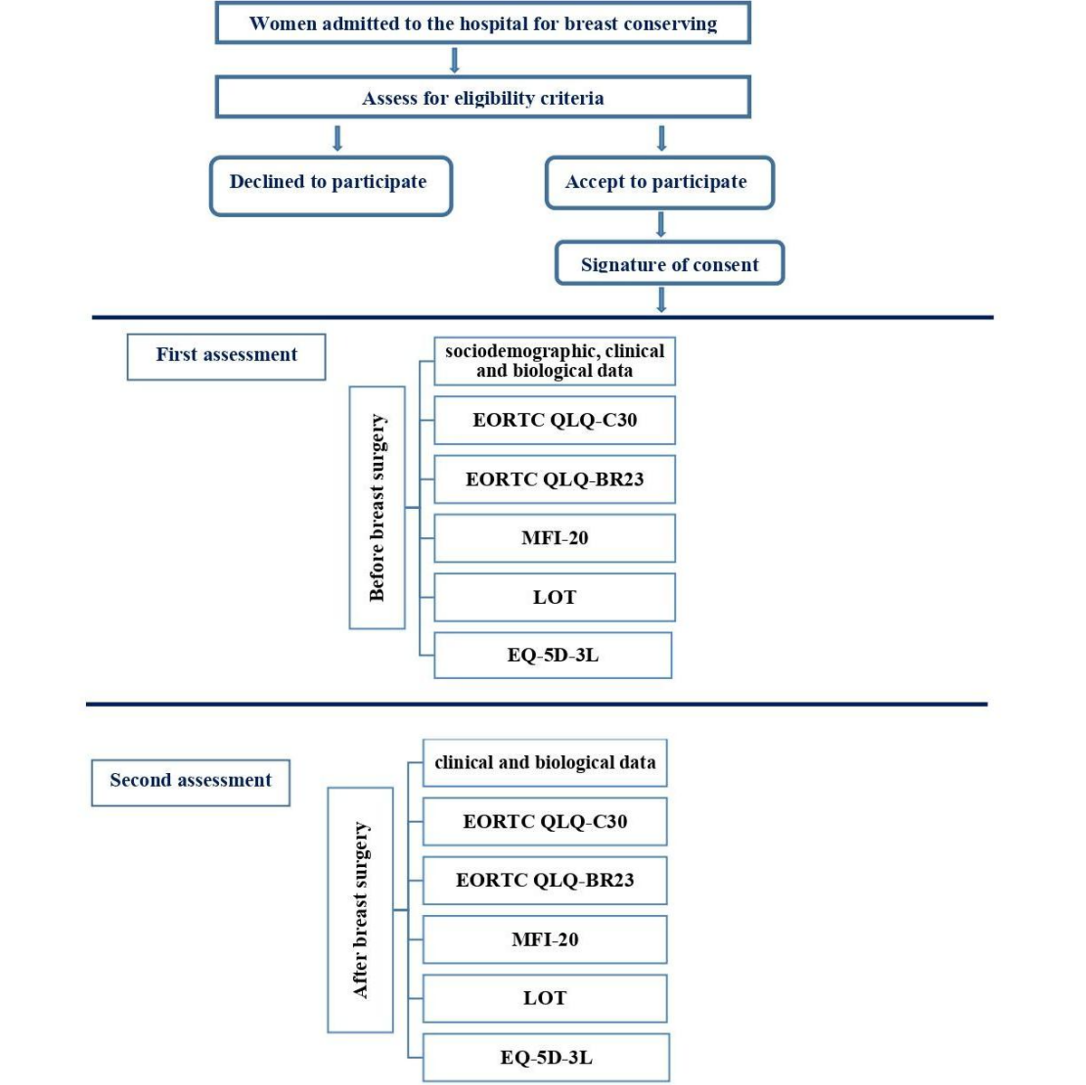
Study flowchart. EORTC QLQ-BR23: European Organization for Research and Treatment of Cancer Quality of Life breast cancer–specific Questionnaire; EORTC QLQ-C30: European Organization for Research and Treatment of Cancer Quality of Life cancer-specific Questionnaire; EQ-5D-5L: European Quality of Life group 5-Dimensional questionnaire–5 Level version; LOT: Life Orientation Test; MFI-20: Multidimensional Fatigue Inventory.

After an explanation of the study, the principal researcher will answer the participants’ questions and present the consent form for signature. Patients will complete the questionnaires during 2 periods: the first assessment will be done on the admission day, and the second period is 1 day after the surgery (Figure 1). The estimated average time spent on assessment will be 30-45 minutes. The patients will complete the following questionnaires:

- The European Organization for Research and Treatment of Cancer Quality of Life cancer-specific questionnaire (EORTC QLQ-C30) to assess HRQoL [31]
- The European Organization for Research and Treatment of Cancer (EORTC) Quality of Life breast cancer–specific questionnaire (QLQ-BR23) to assess HRQoL
- The Multidimensional Fatigue Inventory (MFI-20) to assess the multidimensional aspect of fatigue [32]
- The Life Orientation Test (LOT) to assess Optimism and Pessimism [24]
- The European Quality of Life group 5-Dimensional questionnaire–5 Level version (EQ-5D-5L) to assess HRQoL [33]

The study will be conducted in 2 hospitals located in Beirut, namely Rafik Hariri University Hospital and Sahel General Hospital. The duration of the study is estimated at approximately 34 months.

### Ethical Statement

The proposal of the study was approved by the institutional review board of the Rafik Hariri University Hospital in Beirut (reference number: 18.007-Trans-CMO-[OM]) and the ethical committee of Sahel General Hospital. Informed consent was obtained from each participant. All necessary measures to safeguard participants’ anonymity and confidentiality of information were respected.

### Outcome Measures

The primary outcome measure will be a change in the body image dimension of HRQoL. The secondary outcome measures will be changes in the HRQoL, first according to fatigue and second according to optimism and pessimism; the EORTC QLQ-C30 dimension (physical, emotional, social, role, cognitive, pain, nausea or vomiting, constipation, diarrhea, insomnia, dyspnea, and appetite loss); and the EORTC QLQ-BR23 dimension (sexual functioning, sexual enjoyment, future perspective, systemic therapy side effects, breast symptoms, arm symptoms, and upset by hair loss), as summarized in Table 1.

**Table 1:** Outcome measures and source data.

Outcome and measure		Validation in Arabic language	Description	Source of data	Period of assessment	
					Before surgery	After surgery
**HRQoL^a^**						
	EORTC QLQ-C30^b^ [30]	✓^c^	A cancer HRQoL questionnaire that has been widely used in clinical trials and investigations for individual patient with cancer management 30 items Includes 5 function domains: physical, emotional, social, role, cognitive; and 9 symptoms: fatigue, pain, nausea or vomiting, constipation, diarrhea, insomnia, dyspnea and appetite loss and global health or quality-of-life and financial impact Recall period: past week Items 1-28 formed by Likert scale: 1-4 (very much) Items 29-30 formed by Likert scale: 1-7 (excellent)	PRO^d^	✓	✓
	EORTC QLQ-BR23^e^ [40]	✓	A breast cancer–specific module of European Organization for Research and Treatment of Cancer Quality of Life Questionnaire 23 items Includes 8 function domains: body image, sexual functioning, sexual enjoyment, future perspective, systemic therapy side effects, breast symptoms, arm symptoms, and upset by hair loss Recall period: past week Items 1-23 formed by Likert scale: 1-4 (very much)	PRO	✓	✓
	EQ-5D-5L^f^ [33,35,36]	✓	A measure of HRQoL that can be used in a wide range of health conditions and treatments Includes 5 dimensions: mobility, self-care, usual activities, pain or discomfort, anxiety or depression Includes a visual analog scale Such dimensions are evaluated by 3 levels: no problems, some problems, extreme problems	PRO	✓	✓
**Body image**						
	EORTC QLQ-BR23 [40]	✓	—^g^	PRO	✓	✓
**Fatigue**						
	MFI-20^h^ [39]	✓	A scale designed to evaluate the dimensions of fatigue 20 items Includes 5 dimensions: general fatigue, physical fatigue, reduced activity, reduced motivation, mental fatigue Recall period: past 4 weeks Items 1-20 formed by Likert scale: 1-5 (no, it’s not true)	PRO	✓	✓
**Optimism and pessimism**						
	LOT^i^ [41]	✓	A scale was developed to assess individual differences in generalized optimism versus pessimism: 10 items Distinguishing between optimism and pessimism 3 items measure the optimism, and 3 items measure the pessimism Recall period: present Items 2, 5, 6, and 8 are fillers Items 1-10 formed by Likert scale: 1-4 (strongly disagree)	PRO	✓	✓

^a^HRQoL: health-related quality of life.

^b^EORTC QLQ-C30: European Organization for Research and Treatment of Cancer Quality of Life cancer-specific Questionnaire.

^c^Feature present.

^d^PRO: patient-reported outcome.

^e^EORTC QLQ-BR23: European Organization for Research and Treatment of Cancer Quality of Life breast- cancer–specific Questionnaire.

^f^EQ-5D-5L: European Quality of Life group 5-Dimensional questionnaire–5 Level version.

^g^Not available.

^h^MFI-20: Multidimensional Fatigue Inventory.

^i^LOT: Life Orientation Test.

#### Body Image

Body image will be evaluated using the Arabic version of the EORTC QLQ-BR23 [30]. The breast cancer module is designed for patients with different disease stages and treatment modalities [34].

Functional and symptomatic items of the QLQ-BR23 questionnaire will be rated on a 4-level response system from *not at all* (score 1) to *very much* (score 4), whereas the global QoL (Q29 and Q30) will be rated on a 7-point response scale.

#### Health-Related Quality of Life

HRQoL will be evaluated using the Arabic versions of the EORTC QLQ-C30 and its QLQ-BR23 [30] and the Arabic version of the EQ-5D-5L [33,35,36].

The EORTC QLQ-C30 is a cancer-specific HRQoL questionnaire that has been widely used in clinical trials and investigations for individual cancer patient management [31]. It comprises 30 items. Items 1-28 are assessed on a 4-point Likert scale from 1, *not at all,* to 4, *very much*, and items 29 and 30 from 0 to 7. The EORTC QLQ-C30 incorporates 5 functional scales (physical, role, cognitive, emotional, and social), 3 symptom scales (fatigue, pain, and nausea and vomiting), a global health status or QoL scale, and a number of single items assessing additional symptoms commonly reported by patients with cancer (dyspnea, loss of appetite, insomnia, constipation, and diarrhea), and the perceived financial impact of the disease.

For each scale, a score is generated from 0 to 100 according to the recommendations of the EORTC such that a high score will correspond to a high level of global health/QoL health, a high functional level, and a high symptomatic level.

The QLQ-BR23 incorporates 5 multi-item scales to assess systemic therapy side effects, arm symptoms, breast symptoms, body image, and sexual functioning. In addition, single items assess sexual enjoyment, hair loss, and future perspectives [31]. As with the EORTC QLQ-C30, a score is generated per dimension such that a high score will reflect a high functional level and a high symptomatic level [31].

The EQ-5D-5L was also used to assess the patients’ HRQoL levels. The Arabic version was requested from the European Quality of Life group [33]. The EQ-5D-5L descriptive system comprises the same 5 dimensions as the EQ-5D-3L (mobility, self-care, usual activities, pain or discomfort, and anxiety or depression); however, each dimension now has 5 response levels: no problems, slight problems, moderate problems, severe problems, and unable to or extreme problems. The respondent is asked to indicate his or her health state by checking the box next to the most appropriate response level for each of the 5 dimensions [32]. Furthermore, another part of EQ-5D-5L is a visual analog scale, which can be used to assess the self-rated health of respondents using a 100 mm scale with the score ranging from 0 (the worst health you can imagine) to 100 (the best health you can imagine). The Arabic version of EQ-5D-5L has been used and validated elsewhere, and it was requested from the European Quality of Life group [33,35,36].

#### Fatigue

The MFI-20 is a self-report tool that has been used to assess fatigue in patients with a variety of cancers [37,38]. The MFI-20 Arabic version will be used to evaluate the multidimensional aspects of fatigue [39]. The scale comprises items evaluating general fatigue, physical fatigue, reduced activity, reduced motivation, and mental fatigue during the past 4 weeks. Each item is scored on a 5-point Likert scale from 0 (yes, it’s true) to 5 (no, it’s not true) [32], which is a 20-item scale evaluating the dimensions of fatigue.

#### Optimism and Pessimism

The LOT is a 10-item self-report measure; 4 of the items are filler items that are included to disguise (somewhat) the underlying purpose of the test. Of the 6 scored items, 3 are phrased in an optimistic direction and 3 in a pessimistic direction. The respondents indicated the extent to which they agreed with each of the items on a 5-point scale from 0 (strongly disagree) to 4 (strongly agree). The recall period was the present. The total score was calculated by the addition of the optimism raw scores and the inverted pessimism raw scores. Scores range from 0 to 24; higher scores indicate greater optimism, and lower scores indicate lower optimism, often referred to as pessimism [24].

### Statistical Considerations

#### Population Analysis

The primary population of analysis will be all included patients with both questionnaires completed pre- and postsurgery. Secondary analyses will be conducted on all included patients with at least the baseline questionnaire completed.

#### Sample Size

According to the International Agency for Research on Cancer, the number of new breast cancer cases in Lebanon in 2018 was estimated to be 3219, regardless of the stage of the disease [10]. To demonstrate a change of at least 5 points and considering a bilateral type 1 error rate of 5% and a statistical power of 80% as the minimal clinically important difference in the body image score after surgery as compared with the baseline score, with an SD of 20 points, it will be required to include 120 patients (paired *t* test) with both baseline and postsurgery scores available. Indeed, to explore factors associated with change in the body image scale after surgery, a multiple linear regression model will be used. With a sample size of 95 patients, a bilateral type 1 error rate of 5%, and a statistical power of 80%, we will be able to introduce 5 explanatory variables in the model in addition to age as a control variable. To consider 5% of nonexploitable data, a total of 126 patients need to be included.

#### Statistical Analysis

Descriptive statistics will be calculated for all study variables at specified times. The qualitative variables will be expressed using frequencies and percentages, whereas quantitative data will be presented as means and SDs, and demographic and clinical characteristics of patients with breast cancer will be compared using the *t* test for continuous variables and chi-square tests for categorical variables. To determine potential predictors of HRQoL, bivariate associations among candidate predictor variables (demographic information, including age, marital status, family situation, education, employment status, habitation, and medical data, including the type of surgery, pathological tumor size, histological grade of tumor, menopausal status, and hormone receptor status) and HRQoL pre- and post-BCS scores were examined using Pearson correlation coefficients. Significant predictor variables for HRQoL will be included in the multiple regression analyses.

Variables with a P value ≤.20 in the univariate model were eligible for the multivariate model. The collinearity between variables will be tested using univariate analysis. Variables presenting collinearity will not be simultaneously included in the same multivariate model. A stepwise selection approach will be then used to select the final multivariate model. The 95% CI will be calculated, and *R^2^* values will be computed to determine how the data fit the regression model.

Repeated measures analysis of variance (ANOVA) will be used to determine surgery effects on the variables of interest. To evaluate the relationships between body image scores, fatigue scores, optimism and pessimism scores, and HRQoL scores, Spearman correlation coefficients will be calculated for all patients pre- and post-BCS.

The scale scores of the EORTC QLQ-C30 and QLQ-BR23 will be computed as recommended [31]. The minimal clinically important difference will be set at 5 points for each score of the EORTC QLQ-C30 and QLQ-BR23 questionnaires [31,42] and at 10 points for the MFI-20 questionnaire [43]. All the results will be interpreted within the meaning of this minimal clinically important difference, in addition to the statistical significance, to measure the clinical relevance of the results. The percentage of missing questionnaires and missing items for the HRQoL questionnaire will be provided. Only women who provide information pre and after operation could be included for the comparison.

### Analysis of the Primary Outcome

Normally distributed continuous outcomes will be summarized as means and SDs. The rate of missing data at each HRQoL measurement time will be reported.

To compare the main scores of the EORTC QLQ-C30, QLQ-BR23, EQ-5D-5L, MFI-20, and LOT across pre- and postsurgery data, a paired *t* test will be used.

To compare possible differences between pre- and postsurgery outcomes, a one-way ANOVA will be used for quantitative variables.

The factors associated with the change in HRQoL according to body image before BCS will be identified using a multiple regression model. To determine the factors potentially predicting the change in HRQoL according to body image, Pearson correlation coefficients between all the numerical variables collected at inclusion and the body image scores at baseline and after surgery will be calculated.

### Analysis of the Secondary Outcomes

Independent sample *t* tests and ANOVAs will be used to test differences in the mean scores of the main questionnaires used in this study and their subscales (EORTC QLQ-C30, QLQ-BR23, EQ-5D-5L, MFI-20, and LOT) and mean scores of demographic variables (age, residence, employment, and income) and clinical characteristics (comorbidities, BMI, and laboratory values). Pearson correlation between EORTC QLQ-C30 and EQ-5D-5L, MFI-20, and LOT scores and their subscales will be calculated to see which domain has the strongest relationship with QoL after surgery.

All analyses will be calculated with their 95% CIs; statistical significance will be set at P<.05. The collected data will be captured and analyzed using the SPSS software version 26 (IBM Corp).

### Ethics Approval and Consent to Participate

The proposal of study is approved by the institutional review board of the Rafik Hariri University Hospital in Beirut (reference number: 18.007-Trans-CMO-[OM]) and the ethical committee of Sahel General Hospital.

## Results

The study received ethics approval from the institutional review board. Recruitment and enrollment began in January 2018. A total of 120 patients were enrolled in this study. Future outcomes will be published in professional peer-reviewed health-related research journals.

## Discussion

### Principal Findings

Nowadays, Lebanese women in general reflect the importance of gender roles in the Lebanese culture and society; most women are still expected to be homemakers, taking care of their families’ needs, although many of them work outside the house and are breadwinners as much as their male partners [30]. However, most studies show that patients with breast cancer experience physical symptoms and psychosocial distress that adversely affect their HRQoL [44,45]. So far, according to limited data, there is a weakness in studies concerning the impact of HRQoL on Lebanese women with breast cancer [29]. This study will report an important gap in the literature by answering a crucial question regarding the patient-reported outcome of HRQoL after BCS. In this context, we designed a study to evaluate HRQoL changes between pre- and post-BCS and just before initiation of adjuvant therapy in newly diagnosed patients with invasive breast cancer, targeting the body image dimension as the primary outcome of interest.

This study is strengthened by its follow-up nature, allowing us to draw conclusions about causality. However, the questionnaires used were well-validated in Lebanese populations. However, some limitations must be acknowledged. As it is a sample of convenience and not population based, the generalizability of the results to the entire Lebanese population is not possible as data were collected from 2 hospitals only. However, our hospitals are a reference center in Beirut city for the treatment of breast cancer, and our population could be representative of this group of patients. On the other hand, considering the gap in the scientific literature on prospective studies focusing on HRQoL, body image, fatigue, and optimism and pessimism before and after BCS, this study is presented as the first prospective study on HRQoL of breast cancer in Lebanon.

Subgroup analysis will be performed to compare baseline characteristics and will be adjusted in the multivariable analysis considering those variables with significant differences.

### Conclusions

In summary, the findings of this study will help decision makers, physicians, and social workers design a comprehensive program with multidisciplinary components for the management and care of patients with breast cancer in Lebanon.

## Abbreviations

ANOVA: analysis of variance
BCS: breast-conserving surgery
EORTC: European Organization for Research and Treatment of Cancer
EQ-5D-5L: European Quality of Life group 5-Dimensional questionnaire–5 Level version
EORTC QLQ-C30: European Organization for Research and Treatment of Cancer Quality of Life cancer-specific Questionnaire
HRQoL: health-related quality of life
LOT: Life Orientation Test
MFI-20: Multidimensional Fatigue Inventory
QLQ-BR23: Quality of Life breast cancer–specific Questionnaire
QoL: quality of life
